# The Therapeutical Properties of Veratrum Viride

**Published:** 1854-02

**Authors:** 


*The Therapeutical Properties of Veratrum Viride.* By Wesley C. Norwood, M. D.

Dr. Norwood, in this pamphlet, has collected his various writings on the subject of American hellebore, together with the opinions of physicians expressed in letters to him. The pamphlet is distributed, gratuitously, by the druggists who have the tincture on sale. We know nothing experimentally of this drug. Mr. A. I. Mathews can furnish it to those inclined to test it.

